# Impairment of Bone Health in Pediatric Patients with Hemolytic Anemia

**DOI:** 10.1371/journal.pone.0108400

**Published:** 2014-10-09

**Authors:** Michael M. Schündeln, Sarah C. Goretzki, Pia K. Hauffa, Regina Wieland, Jens Bauer, Lena Baeder, Angelika Eggert, Berthold P. Hauffa, Corinna Grasemann

**Affiliations:** 1 Department of Pediatric Hematology and Oncology, Kinderklinik III, Universitätsklinikum-Essen and the University of Duisburg-Essen, Essen, Germany; 2 Department of Pediatric Endocrinology and Diabetology, Kinderklinik II, Universitätsklinikum-Essen and the University of Duisburg-Essen, Essen, Germany; 3 Department of Pediatrics, Division of Oncology and Hematology, Charité – Universitätsmedizin, Berlin, Germany; University of Tokyo, Japan

## Abstract

**Introduction:**

Sickle cell anemia and thalassemia result in impaired bone health in both adults and youths. Children with other types of chronic hemolytic anemia may also display impaired bone health.

**Study Design:**

To assess bone health in pediatric patients with chronic hemolytic anemia, a cross-sectional study was conducted involving 45 patients with different forms of hemolytic anemia (i.e., 17 homozygous sickle cell disease and 14 hereditary spherocytosis patients). Biochemical, radiographic and anamnestic parameters of bone health were assessed.

**Results:**

Vitamin D deficiency with 25 OH-vitamin D serum levels below 20 ng/ml was a common finding (80.5%) in this cohort. Bone pain was present in 31% of patients. Analysis of RANKL, osteoprotegerin (OPG) and osteocalcin levels indicated an alteration in bone modeling with significantly elevated RANKL/OPG ratios (control: 0.08+0.07; patients: 0.26+0.2, *P* = 0.0007). Osteocalcin levels were found to be lower in patients compared with healthy controls (68.5+39.0 ng/ml vs. 118.0+36.6 ng/ml, *P* = 0.0001). Multiple stepwise regression analysis revealed a significant (*P*<0.025) influence of LDH (*partial r^2^ = 0.29*), diagnosis of hemolytic anemia (*partial r^2^ = 0.05*) and age (*partial r^2^* = 0.03) on osteocalcin levels. Patients with homozygous sickle cell anemia were more frequently and more severely affected by impaired bone health than patients with hereditary spherocytosis.

**Conclusion:**

Bone health is impaired in pediatric patients with hemolytic anemia. In addition to endocrine alterations, an imbalance in the RANKL/OPG system and low levels of osteocalcin may contribute to this impairment.

## Introduction

Chronic hemolytic anemia is a rare condition characterized by an abnormal breakdown of erythrocytes. A plethora of pathogenic factors can promote the development of chronic hemolysis (reviewed by Dhaliwal et al. [1]). Intrinsic hemolytic anemia can be subclassified as membranopathy (e.g., spherocytosis), hemoglobinopathy (e.g., sickle cell disease) or enzymopathy (e.g., pyruvate kinase deficiency). In contrast, extrinsic hemolytic anemias are caused by conditions such as autoimmunity or microangiopathy.

Impaired bone health has been described in patients with sickle cell disease and thalassemia. These patients display altered parameters of bone metabolism and bone mineral density [2]–[5].

Vitamin D and growth hormone deficiency, chronic transfusion, iron toxicity, as well as diabetes and other endocrine pathologies have been identified to promote osseous pathology [6]. Certain lifestyle parameters, such as smoking or lack of physical activity, also have a detrimental impact on bone health [6].

Analysis of a murine model of hemolytic anemia (*plasmodium chabaudi* infection or phenylhydrazine treatment) has indicated that anemia itself may be detrimental to bone health. Moreau et al. [7] have demonstrated that mice with severe acute hemolytic stress displayed reduced bone mineral density and decreased levels of the bone formation marker osteocalcin.

Recently, a number of reports have emerged suggesting an imbalance in bone remodeling in adult patients with sickle cell disease or thalassemia, as indicated by an elevated RANKL/OPG ratio or increased TRAP5b activity [8]–[10].

However, to our knowledge, few data are available regarding bone health in pediatric patients, especially for patients with rare forms of hemolytic anemia, such as hereditary spherocytosis or glucose-6-phosphate deficiency.

To assess pediatric bone health in these conditions, we conducted a cross-sectional study of bone health in pediatric patients with hemolytic anemia.

## Results

### Bone health in pediatric patients with hemolytic anemia

The descriptive statistics of the 45 patients and controls are shown in Tables 1 and 2. Male and female patients did not significantly differ with respect to biochemical or clinical findings (data not shown).

**Table 1:** Clinical characteristics, parameters of disease activity and bone health.

	All patients (n = 45)	HbSS (n = 17)	Spherocytosis (n = 14)	Healthy controls (n = 14)	P-Value
**Female/male**	23/22	9/8	7/7	7/8	
**Age (years)**	9.8±4.4 (1.1–18.4)	9.3±4.4 (2.2–18.38)	10.45±4.4 (2.5–17.9)	10.3±3.6 (0.8–14.7)	0.45
**BMI SDS**	0.01±1.1 (−3.1–2.33)	−0.11±1.4 (−3.10–2.33)	0.22±0.76 (−1.58–1.44)	−0.16±1.17 (−2.05–1.83)	0.32
**Pubic hair stage SDS**	−0.15±0.69 (−2.24–1.18)	−0.28±0.64 (−1.73–0.66) 9	0.14±0.42 (−0.64–0.87) 8	0.10±1.0 (−1.4–1.6), 10	0.38
**TV/Breast stage SDS**	−0.17±0.93 (−2.73–1.94)	−0.20±0.7 (−1.37–0.63) 8	0.33±1.0 (−1.34–1.94) 8	0.21±1.2(−1.35–1.82), 10	0.43
**Height SDS**	−0.27±0.87 (−2.65–1.46)	−0.17±0.9 (−1.81–1.45)	0.16±0.6 (−0.79–1.23)	−0.09±2.3 (−3.79–3.74)	0.39
**LDH (U/l)**	414±188.6 (164–) 44	569.4±138.4 (386–979) 17	283.1±42.4 (213–362) 13	214.5±36.4 (168–255) 6	<0.0001
**Bili (mg/dl)**	2.53±1.64 (0.2–6.9) 44	3.08±1.1 (1.5–5.5)	3.03±1.9 (1.0–6.9) 13	0.53±0.3 (0.3–1.1) 7	<0.001
**Retic (^0^/_00_)**	136.1±107.4 (9–352) 39	195±93.6 (69–352) 15	135±108 (9.0–340) 14	NA	*0.08*
**25-OH Vit D (ng/ml)**	12.6±7.9 (1–30.2) 44	9.3±7.4 (1–25.2)	19.1±5.7 (12.8–30.2) 14	10.8±8.8 (1–20.6) 4	0.004
**1,25-OH Vit D (pg/ml)**	48.4±19.8 (18–118) 37	46.6±13.7 (18–68) 15	51.5±30.2 (23–118) 11	NA	*0.73*
**SAP (U/l)**	216.2±106.7 (47–646) 44	212.7±84.4 (67–417)	173.2±78.8 (47–311) 13	231.4±57.12 (171–332) 7	*0.25*
**BAP (U/l)**	131.4±85.0 (26.4–531.4) 44	124.6±55.1 (41.5–261)	111.9±62.7 (26.4–247) 11	NA	*0.002*
**PTH (pg/ml)**	47.1±34.8 (17.3–239.6) 44	43.7±19.3 (23.0–89.7)	37.6±15.6 (17.3–72.1) 14	42.2±6.44 (35.9–50.0) 4	0.45
**NTX (nmolBCE/nmol crea)**	715.2±648.9 (117–1994) 10	952.5±732 (163–1994) 6	117 (N = 1)	NA	NA
**DPD (µg/g crea)**	157.0±77.9 (20–310) 29	187.2±81.4 (63–310) 12	124.1±70.8 (20–216) 10	NA	*0.11*
**Ca:Crea (mg/mg)**	0.07±0.07 (0.01–0.31) 37	0.05±0.04 (0.004–0.16) 16	0.08±0.07 (0.01–0.25) 10	NA	0.24
**Osteocalcin (ng/ml)**	68.5±39.0 (17.5–204) 33	45.6±17.6 (17.5–76.6) 12	90.0±46.7 (37.5–204.3) 13	115.3±35.2 (72.6–186.1) 13	<0.0001
**IGF-1 SDS**	−0.62±1.2 (−3.6–1.7) 39	−1.04±1.37 (−3.6–0.9) 14	−0.36±1.16 (−2.0–1.7) 14	1.3±1.3 (−0.2–3.7) 12	<0.001
**RANKL (pmol/l)**	0.87±0.64 (0.00–2.77) 17	1.18±0.72 (0.44–2.77) 8	0.57±0.47 (0.0–1.45) 8	0.29±0.26 (0.00–0.92) 14	0.002
**OPG (pmol/l)**	3.29±0.55 (2.3–4.4) 17	3.63±0.45 (2.9–4.4) 8	2.93±0.46 (2.3–3.5) 8	3.48±0.64 (2.80–4.70) 14	0.04
**DXA (Z-Score)**	−0.74±1.0 (−2.5–0.7) 14	−0.6±1.04 (−2.2–0.7) 9	−0.7 (N = 1)	NA	NA

Mean ± SD, (range) are displayed. Followed by the number of patients examined if different from total number. (Pubic hair stage SDS (PH SDS), testicular volume/breast development stage SDS (TV/breast stage SDS), Lactate dehydrogenase (LDH), bilirubin (bili), reticulocytes (retic), 25-OH vitamin D (25-OH Vit D), 1,25-(OH)_2_ vitamin D (1,25-OH Vit D), serum alkaline phosphatase (SAP), bone alkaline phosphatase (BAP), parathyroid hormone (PTH), urinary N-terminal telopeptide (NTX), urinary deoxypyridinoline (DPD), urinary calcium:creatinine ratio (Ca:Crea), osteocalcin, insulin-like growth factor 1 SDS (IGF-1 SDS), receptor activator of nuclear factor kappa-B (RANKL), osteoprotegerin (OPG) and dual-energy X-ray absorptiometry (DXA) Z-Score) were assessed. P-values refer to Kruskal Vallis test (HBSS vs Spherocytosis vs Healthy controls) if values are available for all 3 groups, or to Wilcoxon-two-sample test if values are available for HBSS and Spherocytosis only (in cursive).

**Table 2:** Altered parameters of bone metabolism [altered bone specific alkaline phosphatase (BAP) or alkaline phosphatase (SAP), elevated parathyroid hormone (PTH), altered urinary N-terminal telopeptide (NTX) or urinary deoxypyridinoline (DPD)] and presence of bone pain in patients.

	All Patients	HbSS	Spherocytosis
**25-OH Vitamin D<20 ng/ml**	80.5% (33/41)	86.7% (13/15)	61.5% (8/13)
**25-OH Vitamin D<10 ng/ml**	50% (20/41)	80% (12/15)	0% (0/13)
**BAP/SAP altered**	11.6% (4/43)	13% (2/15)	0% (0/13)
**PTH elevated**	22.5% (9/40)	21.4% (3/14)	15.3% (2/13)
**NTX/DPD altered**	8.6% (3/35)	16.7% (2/12)	0% (0/12)
**Regular back pain**	32.4% (12/37)	41.7% (5/12)	15.3% (2/13)
**Knee pain with exercise**	19.4% (7/36)	18.2% (2/11)	7.7.% (1/13)

Percentage and fraction (in brackets) of affected patients are displayed.

A vitamin D deficiency (serum 25-OH vitamin D<20 ng/ml) was present in 79.6% of the patients, 43.2% of patients displayed severe vitamin D deficiency (serum 25-OH vitamin D<10 ng/ml). The mean serum 25-OH vitamin D level was 12±7.6 (range: 1–30.2) ng/ml. Serum 1, 25-(OH)_2_ vitamin D levels were largely within the normal range, 49.6±19.8 (range: 18–118) ng/l. Mean serum PTH levels were in the upper normal range, 49.1±35.9 (range: 17.3–239.6) pg/ml. However, hyperparathyroidism developed in more than 20% of the children. For the complete results of parameters relevant to bone metabolism refer to Tables 1 and 2.

In the subgroup of patients in whom bone mineral density were assessed via dual-energy X-ray absorptiometry (DXA) scan, the mean Z-score was negative (−0.74±1 (−2.5 to 0.7, n = 14), although most readings were within the normal range. Osteopenia (Z-score <−2) was detected in 14% of the patients screened.

The mean calcium:creatinine ratio in spot urine was low, 0.07±0.07 (0.01–0.32) mg/mg.

**Figure 1:**
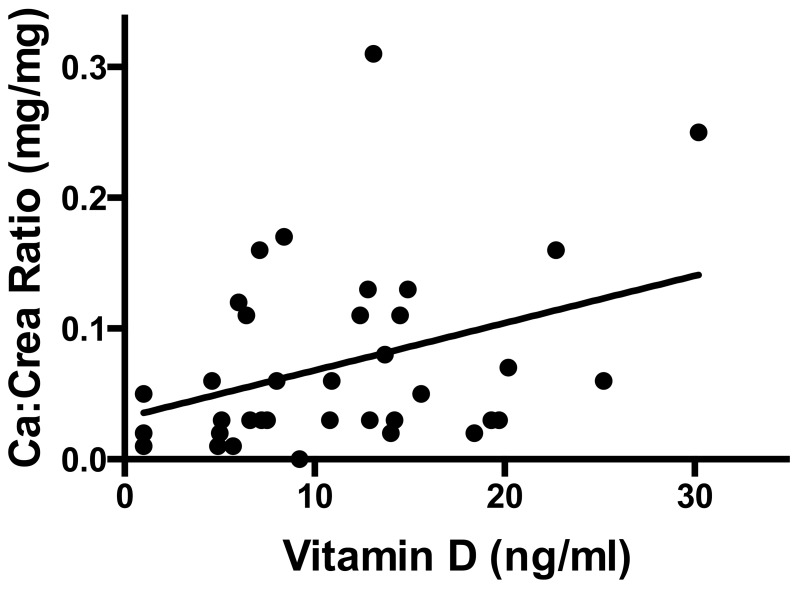
Serum levels of 25-OH vitamin D levels positively correlate with calcium to creatinine ratio in urine (*P* = 0.03, r = 0.36) in patients with hemolytic anemia. The predicted values based on bivariate regression are indicated as solid line.

The calcium:creatine ratio in urine positively correlated with 25-OH vitamin D levels in patients with hemolytic anemia (r = 0.36, *P* = 0.03, Figure 1).

Mean osteocalcin levels were 75±51.1 (17.5–247 ng/ml) as compared to 115.3±35.2 (72.6–186.1 ng/ml) in healthy controls. 45% of all patients had an osteocalcin serum level below the 25^th^ percentile for age, and approximately 16% of patients were below the 2.5^th^ percentile. IGF-1 SDS values were negative in most of the population.

Patient values for SAP, BAP, NTX, DPD, leptin and IGF-1 were compared with age appropriate normative values; the results are displayed in Table 1.

A strong positive correlation between SAP and BAP levels (r = 0.94, *P*<0.0001) and between SAP and PTH levels (r = 0.69, *P*<0.0001) was observed. A positive correlation was found between NTX and BAP levels (r = 0.89, *P* = 0.0012, n = 9) and between NTX and RANK/OPG levels (r = 0.90, *P* = 0.04, n = 5) in a subgroup of patients in whom both parameters were measured.

Vitamin D uptake levels, as assessed via questionnaire, were low in all patients, although they were relatively higher in the HbSS group (196 U/d vs. 84 U/d). Mean calcium intake/d was 855±438 (range: 305–1984) mg. Average screen time was 2.1±1.5 (range: 0–5) h/d, and average daily activity was 3.4 h/d. Furthermore, 12% of the patients had experienced long bone fractures.

**Figure 2:**
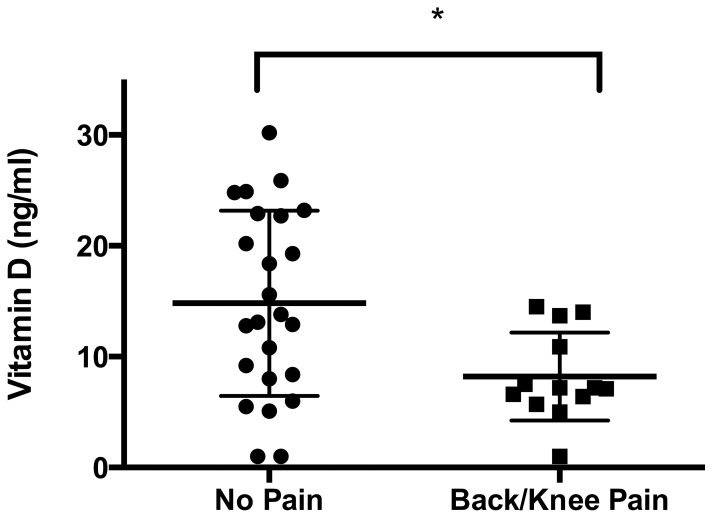
Serum 25-OH vitamin D levels are significantly lower in patients who report bone pain (back pain and/or knee pain with exercise) than in patients without reported bone pain (left). Statistically significant differences between the groups, determined via Mann-Whitney test, are indicated with asterisks (*: *P*<0.05).

25-OH vitamin D levels were significantly lower in patients with frequent back pain or knee pain associated with exercise, than in patients without bone pain (15.6±1.6 vs. 9.9±1.6 ng/ml, *P* = 0.027, Figure 2). Serum levels of alkaline phosphatase were higher in patients with self-reported knee pain (279.8+76.1 U/l) than in patients without a history of pain (179.5+64.9 U/l, *P* = 0.0016).

### Comparison of bone health in patients with homozygous sickle cell disease and spherocytosis

The two largest subgroups in this cohort were patients with homozygous sickle cell anemia (HbSS, n = 17) or spherocytosis (n = 14). These groups did not differ in terms of age, sex or pubertal status (Table 1).

Serum levels of 25-OH vitamin D in patients with HbSS were approximately half of those in patients with spherocytos is. Severe vitamin D deficiency (<10 ng/ml) was not detected in children with spherocytosis, but in 80% of the patients with HbSS.

Hyperparathyroidism was observed in 22% of patients with HbSS, while it was found in 14% of patients with spherocytosis.

**Figure 3:**
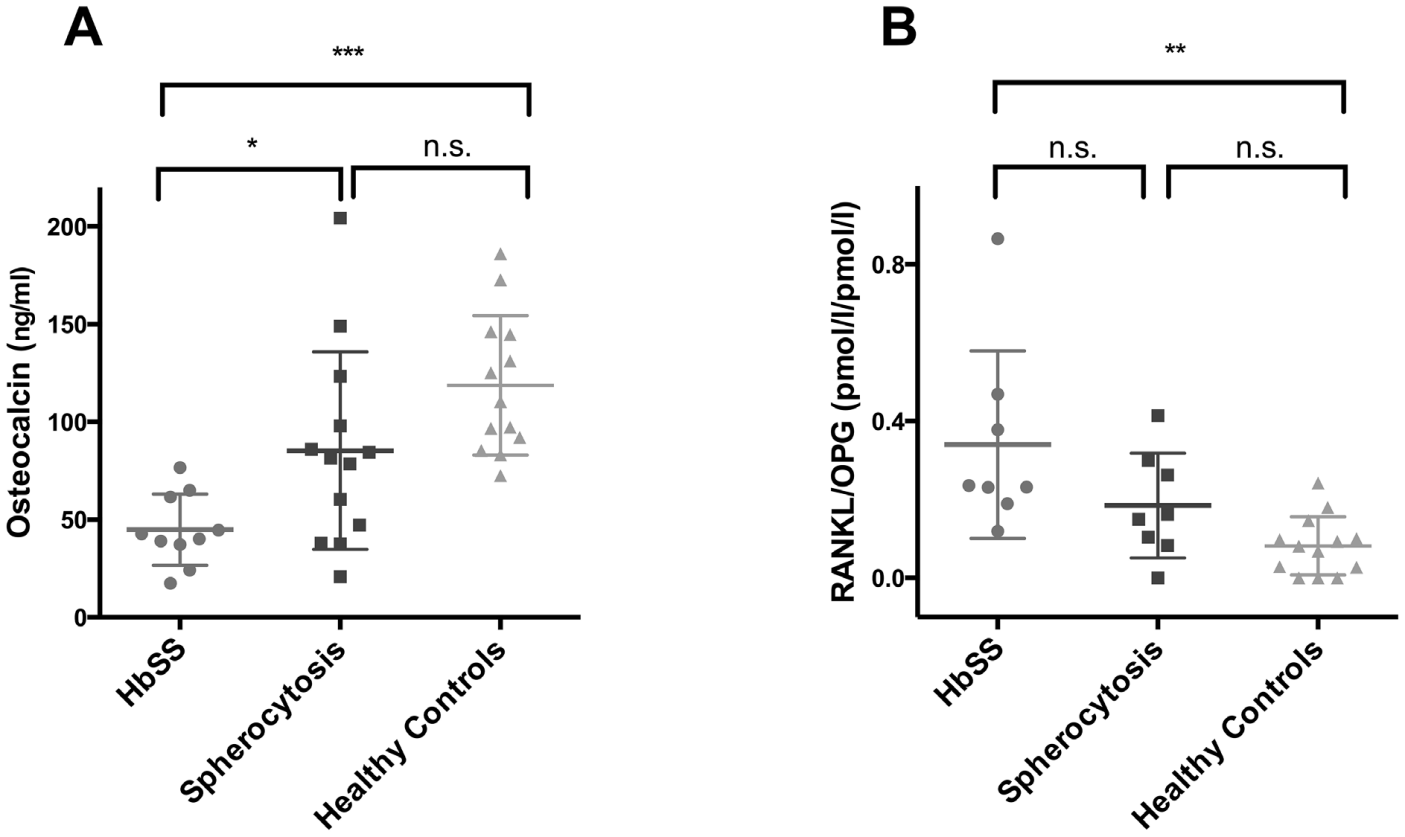
Parameters of bone remodeling are altered in patients with sickle cell disease. Distribution (lines indicate mean and standardvariation) of A) osteocalcin and B) RANKL/OPG ratio in patients with sickle cell disease (HbSS. grey circles) and in patients with spherocytosis (Spherocytosis, black squares) compared to age- and sex-matched healthy controls (Healthy controls, lightgrey triangles) Statistically significant differences between the groups (as assessed by Mann Whithey-U test with Bonferroni-Holm correction for multiple testing) are indicated with asterisks (*: P<0.05, **: P<0.01, ***: P<0.001).

Osteocalcin levels differed significantly between patients with sickle cell disease, spherocytosis and healthy controls (*P*<0.0001, Kruskal-Wallis test). Patients with sickle cell disease displayed the lowest levels of osteocalcin (45.6±17.6 ng/ml), which were significantly lower than those observed in healthy controls (115.3±35.2 ng/ml), (*P*<0.001, Mann-Whitney-U test with Bonferroni-Holm correction for multiple testing) and in patients with spherocytosis (90.0±46.7 ng/ml), (*P* = 0.015, Mann-Whitney test with Bonferroni-Holm correction for multiple testing) Figure 3.

Serum levels of RANKL were significantly higher in patients with sickle cell disease than in controls (1.18±0.72 pmol/l vs. 0.29±0.26 pmol/l, *P* = 0.0002), whereas levels of osteoprotegerin (OPG) were not altered in these patients. Serum OPG levels were significantly reduced in spherocytosis patients (2.9±0.47 pmol/l) compared with sickle cell disease patients (3.6±0.4 pmol/l, *P* = 0.006). There was a trend showing lower levels of OPG in patients with spherocytosis compared to controls (3.5±0.6 pmol/l, *NS*, Table 1).

The RANKL/OPG ratio was significantly different between patients with sickle cell disease, spherocytosis and healthy controls (*P* = 0.0021, Kruskal-Wallis test). Patients with sickle cell disease displayed the highest ratios for RANKL/OPG (0.34±0.24), which were significantly higher than those observed in healthy controls (0.08±0.07), (*P* = 0.006, Mann-Whitney-U test with Bonferroni-Holm correction for multiple testing). RANKL/OPG ratios in patients with spherocytosis were not significantly different from controls or sickle cell patients. Figure 3.

In a multiple stepwise regression analysis, in which the influence of the variables age, sex, diagnosis (healthy control, HbSS or spherocytosis) and LDH was tested on the variables osteocalcin or RANKL/OPG ratio, a significant effect was detected:

Osteocalcin levels were influenced by LDH (partial r^2^ = 0.29, *P* = 0.03), diagnostic group (partial r^2^ = 0.05, *P* = 0.31) and age (partial r^2^ = 0.03, *P* = 0.41). No influence of gender on osteocalcin levels was found.

RANKL/OPG levels were influenced by age (partial r^2^ = 0.3, *P* = 0.24), LDH (partial r^2^ = 0.11, *P* = 0.13), sex (partial r^2^ = 0.04, *P* = 0.37) and diagnosis (partial r^2^ = 0.03, *P* = 0.46).

## Discussion

### Bone health in pediatric patients with hemolytic anemia

In our tertiary center, pediatric patients with hemolytic anemia displayed a high prevalence of vitamin D deficiency. In this group, patients with sickle cell disease were most frequently affected by vitamin D deficiency and displayed the lowest 25-OH vitamin D levels.

As vitamin D is a major regulator of calcium homeostasis, severe vitamin D deficiency can be accompanied by low calcium stores and secondary hyperparathyroidism.

We assessed urinary calcium excretion levels in spot urine samples to estimate overall body calcium balance and found a significant positive correlation between the calcium:creatinine ratios and 25 OH-Vitamin D levels. Patients with low 25 OH-Vitamin D levels also displayed lower calcium:creatinine ratios, thereby indicating a poor total calcium balance.

Approximately half of the patients with severe vitamin D deficiency developed secondary hyperparathyroidism. These data suggest that vitamin D deficiency is not only a common finding, as previously reported in adult [11] and pediatric [12] patients with sickle cell disease, but it should be treated to avoid secondary damage to the skeletal system.

Amongst other hormones, IGF-1 exhibits positive effects on bone formation (reviewed by Guntur and Rosen [13]). IGF-1 levels in this cohort of patients were subnormal. Low IGF-1 levels contributing to impaired bone health have previously been reported for HbSS [14] and thalassemia [15]. However, this represents the first report of low IGF-1 levels among spherocytosis patients.

It is relatively difficult to assess bone health, especially in a pediatric population. Bone mineral denisity (BMD) assessed via bone densitometry must be corrected for age and height appropriate standards. Reduced BMD below the 2^nd^ standard deviation is probably a late and alarming sign of impaired bone health. As BMD assessment necessitates exposure to x-ray radiation, it was evaluated exclusively in children with suspected bone health impairment in this cohort. The average Z-Score was low with - 0.74. This value is probably biased towards lower values due to the above mentioned criteria for examination, however, it has been shown that reduced BMD is a common finding in pediatric [12] and adult [16] sickle cell disease patients.

Frequent back pain and knee pain after exercise can be indicative of impaired bone health. We aimed to determine whether self-reported bone pain and biochemical parameters of bone metabolism were linked in this cohort of patients.

Surprisingly, patients who reported bone pain displayed significantly lower levels of 25 OH-vitamin D. We also found that recurring knee pain was associated with higher serum alkaline phosphatase levels in patients with hemolytic anemia.

An association between vitamin D deficiency and clinical features of poor musculoskeletal health and especially self-reported chronic pain has previously been reported [17]. In a follow up study, the same group has suggested that supplementation of vitamin D alleviates chronic pain in sickle cell disease patients [18].

### Comparison of bone health in patients with homozygous sickle cell disease and spherocytosis

Comparing the largest subgroups of this cohort, patients with sickle cell anemia are significantly more likely to exhibit severe vitamin D deficiency (<10 ng/ml) than patients with spherocytosis, despite slightly higher intake levels of vitamin D3. It is feasible that different food preferences or different nutritional recommendations by the primary care physicians result in the difference in vitamin D3 intake levels. However, even in patients with sickle cell disease vitamin D3 intake levels were only ¼ of the recommended daily intake of 800 IU in Germany.

Both bone formation and bone resorption are essential for physiological bone modeling and remodeling.

In our study, patients with hemolytic anemia displayed significantly lower levels of osteocalcin (a marker of bone formation) than the healthy control group. This has been previously described in pediatric patients with β-thalassemia and sickle cell disease [3], [19] and may reflect an intrinsic deficit in bone formation in patients with hemolytic anemia.

Patients with hemolytic anemia also displayed a significantly higher RANKL/OPG ratio compared with the control group. Binding of RANKL to its receptor results in osteoclast differentiation and stimulation, while OPG serves as a circulating inactivator of RANKL.

To further analyze these differences we have performed a nonparametric one way ANOVA which detected significant differences in osteocalcin and RANK/OPG levels between patients with sickle cell disease, patients with spherocytosis and healthy controls. Post-hoc analysis revealed significantly lower levels of osteocalcin in patients with sickle cell disease, than in any of the other groups. Conversely, RANK/OPG ratios were significantly higher in patients with sickle cell disease than in healthy controls.

Since sickle cell disease, more so than spherocytosis, is a state of chronic inflammation [20], this fact may contribute to the activation of the RANK pathway and result in activation of osteoclasts. In adults with sickle cell disease, Nouraie and colleagues [8] have reported increased levels of TRAP5b, a marker of osteoclast activity.

It is also feasible that severe hemolysis may be responsible for the more pronounced impairment of bone health in patients with sickle cell disease. We found a significant influence of LDH serum levels on Osteocalcin and RANK/OPG levels in a multiple stepwise regression model, indicating that disease activity is relevant for bone metabolism.

### Limitations of the study

The main limitation of the current study is the small size and heterogeneous nature of the cohort. Due to the small number of patients, for some of the diagnoses, it was not possible to perform a more detailed analysis of subgroups other than sickle cell disease and spherocytosis.

## Material and Methods

### Patients

The study was performed in the Hematology-Oncology outpatient clinic of the Children's Hospital, University of Duisburg-Essen, Germany, between August 2012 and August 2013. A total of 45 patients (24 females, 21 males) with hemolytic anemia were recruited during regular visits after informed consent was obtained from the patients and parents.

The study (German clinical trials register number 00003636) was performed in accordance with the ethical principles of the Declaration of Helsinki and was approved by the ‘Research Ethics Committee of the Medical Faculty, University of Duisburg-Essen’ (12-4966-BO). Patients were included after written informed consent was obtained from patients and caregivers.

In a subgroup of patients, serum levels of osteocalcin (OC), receptor activator of nuclear factor kappa-B ligand (RANKL) and osteoprotegerin (OPG) were assessed and compared with healthy, age-matched controls. The group of healthy controls consisted of children who were examined by the endocrine service for suspected endocrine dysfunction. Parents and children of the healthy control group gave written informed consent to participate in the study.

Patients with the following diagnoses were included in the study: homozygous sickle cell anemia (HbSS, n = 17), sickle hemoglobin C disease (HbSC, n = 2), β-thalassemia major (n = 6), β-thalassemia minor (n = 1), hereditary spherocytosis (n = 14), glucose-6-phosphate deficiency (n = 2), paroxysmal nocturnal hemoglobinuria (PNH, n = 1) and hemolytic anemia of unknown origin (n = 2). Four of the patients with β-thalassemia major had undergone allogeneic bone marrow transplantation. For patient characteristics refer to Table 1.

### Laboratory tests

The following biochemical parameters of disease activity, growth, pubertal status, bone turnover and vitamin D metabolism were assessed in serum or plasma samples: lactate dehydrogenase, LDH (U/l); total bilirubin (mg/dl); reticulocytes (^0^/_00_); 25-OH vitamin D (ng/ml); 1,25-(OH)_2_ vitamin D (pg/ml); serum alkaline phosphatase, SAP (U/l); bone specific alkaline phosphatase, BAP (U/l);); parathyroid hormone, PTH (pg/ml); osteocalcin, OC (ng/ml); insulin-like growth factor-1, IGF-1 (ng/ml); RANKL (pmol/l) and osteoprotegerin, OPG (pmol/l).

Additionally, N-terminal telopeptide, NTX (nmol bone collagen equivalent (BCE)/mmol creatinine) and deoxypyridinoline, DPD (µg/g creatinine), as markers of bone resorption, as well as the calcium:creatinine ratio (mg/mg) were assessed in spot urine samples. Serum IGF-1 levels were expressed in SDS values, according to age and sex, based on the data of Blum and Breier [21]. For calculations of IGF-1 SDS the software tool “SDS-Easy”, (Mediagnost, Reutlingen, Germany) was used, while serum leptin levels were expressed in SDS values according to sex, BMI and Tanner stage [22].

### Bone densitometry

Bone mineral density (BMD) was examined via dual-energy X-ray absorptiometry (DXA) (Lunar Prodigy, GE-Healthcare, Madison, WI, USA) in a small subgroup of patients. BMD was assessed at the lumbar spine (L1–L4; anteroposterior view) and the left femoral neck. Z -scores were calculated for the lumbar spine measurements based on normative values for the corresponding age [23]. A single investigator blinded to the clinical status of the patients was responsible for all BMD measurements.

### Clinical parameters and questionnaire

During regular visits in the outpatient clinic, a physical exam was performed assessing patient height, weight and pubertal staging according to Tanner stage.

Standing height was measured using a wall-mounted stadiometer (Ulmer Stadiometer, Busse Design, Elchingen, Germany) to the nearest mm. Weight was recorded to the nearest 0.1 kg using a digital scale (Seca, Hamburg, Germany). BMI was calculated from these data using the formula weight (kg)/(height^2^) (m^2^). The measurements were transformed into SDS (standard deviation score) based on a reference data set for German children [24]. An experienced pediatrician assessed the pubertal development according to the Tanner stages. Testicular volume was assessed using a Prader orchidometer. Pubertal status data were then transformed into SDS based on the data of Mul et al. Conversion of pubertal stages into SDS was performed using the web application (http://vps.stefvanbuuren.nl/puberty/) (accessed 2014 July 3) by van Buuren and Ooms [25].

Patients were asked to complete a standardized questionnaire regarding vitamin D and calcium intake, nutritional supplements, screen hours per day and hours of physical activity per day. Specifically, patients and parents were asked to provide data on the average amounts of: milk, sparkling water (and type of sparkling water), cereal (and type of cereal), cheese slices, yoghurts, seeds and fish (including type of fish) per week.

Vitamin D intake was calculated according to data charts, published by Heseker [26] and Elmadfa et al. [27].

Additionally, patients were interrogated regarding fracture history and the presence of frequent back pain or knee pain associated with exercise. The number of transfusions undergone during the two years prior to the study visit was obtained via chart review.

### Statistics

Data were tested for normal distribution using the Shapiro-Wilk test. The assumption of a normal distribution was rejected at an alpha <0.1. Since a normal distribution could not be assumed for most parameters, differences between diagnostic groups (healthy controls, HbSS or spherocytosis) were tested by the Kruskal-Wallis test. When an overall difference was found, post hoc analysis was carried out by pairwise comparison of groups using the Mann -Whitney-U test, correcting for multiple testing with the Bonferroni-Holm method. The influence of age, sex, diagnosis (healthy controls, HbSS or spherocytosis) and LDH on bone markers was assessed by multiple stepwise regression analysis. Variables accepted in the model were significant at the 0.5000 level.

Associations between single variables were described by the Spearman correlation coefficient. Differences between group means and reference cohorts were assessed by the Mann-Whitney-U test. Statistical significance was assumed at *P*<0.05. Data are given as mean ±1 SD and range. Data analysis was performed using SAS statistical software (SAS System, release 9.4, SAS Institute Inc., Carey, NC, USA, 2008).

## Conclusions

With this study, we demonstrate that children with hemolytic anemia share features of impaired bone health. These observations provide new insight regarding such patients and support the findings of comparable reports on bone health in adult and pediatric patients with sickle cell disease and thalassemia [6].

Patients with sickle cell disease display more severe alterations of bone health than patients with spherocytosis. Therefore, the pathogenesis and severity of hemolysis may be a critical factor in determining the phenotype of bone pathology.

This report highlights the need for further studies of bone health in patients with hemolytic disease. Our findings also suggest that the paradigm should be modified in that all such patients should be tested regarding bone health.
